# The sense of stopping migraine prophylaxis

**DOI:** 10.1186/s10194-023-01539-8

**Published:** 2023-02-16

**Authors:** Linda Al-Hassany, Hannah S. Lyons, Deirdre M. Boucherie, Fatemeh Farham, Kristin S. Lange, Karol Marschollek, Dilara Onan, Umberto Pensato, Elisabeth Storch, Angelo Torrente, Marta Waliszewska-Prosół, Uwe Reuter, on behalf of the European Headache Federation School of Advanced Studies (EHF-SAS)

**Affiliations:** 1 Department of Internal Medicine, Division of Vascular Medicine and Pharmacology, Erasmus MC University Medical Center, Rotterdam, the Netherlands; 2 Institute of Metabolism and Systems Research, College of Medical and Dental Sciences, University of Birmingham, Birmingham, UK; 3 Department of Headache, Iranian Centre of Neurological Researchers, Neuroscience Institute, Tehran University of Medical Sciences, Tehran, Iran; 4 Department of Neurology, Charité - Universitätsmedizin Berlin, Charitéplatz 1, 10117 Berlin, Germany; 5 Department of Neurology, Wroclaw Medical University, Wrocław, Poland; 6 Spine Health Unit, Faculty of Physical Therapy and Rehabilitation, Hacettepe University, Ankara, Turkey; 7 Department of Clinical and Molecular Medicine, Sapienza University, Rome, Italy; 8 Neurology and Stroke Unit, IRCCS Humanitas Research Hospital, Rozzano, Milan, Italy; 9 Humanitas University, Pieve Emanuale, Milan, Italy; 10 Department of Biomedicine, Neurosciences and Advanced Diagnostics, University of Palermo, Palermo, Italy; 11 Universitätsmedizin Greifswald, Greifswald, Germany

**Keywords:** Migraine, Preventive treatment, Prophylaxis, Stopping rules

## Abstract

**Introduction:**

Migraine prophylactic therapy has changed over recent years with the development and approval of monoclonal antibodies (mAbs) targeting the calcitonin gene-related peptide (CGRP) pathway. As new therapies emerged, leading headache societies have been providing guidelines on the initiation and escalation of such therapies. However, there is a lack of robust evidence looking at the duration of successful prophylaxis and the effects of therapy discontinuation. In this narrative review we explore both the biological and clinical rationale for prophylactic therapy discontinuation to provide a basis for clinical decision-making.

**Methods:**

Three different literature search strategies were conducted for this narrative review. These include i) stopping rules in comorbidities of migraine in which overlapping preventives are prescribed, notably depression and epilepsy; ii) stopping rules of oral treatment and botox; iii) stopping rules of antibodies targeting the CGRP (receptor). Keywords were utilized in the following databases: Embase, Medline ALL, Web of Science Core collection, Cochran Central Register of Controlled Trials, and Google Scholar.

**Discussion:**

Reasons to guide decision-making in stopping prophylactic migraine therapies include adverse events, efficacy failure, drug holiday following long-term administration, and patient-specific reasons. Certain guidelines contain both positive and negative stopping rules. Following withdrawal of migraine prophylaxis, migraine burden may return to pre-treatment level, remain unchanged, or lie somewhere in-between. The current suggestion to discontinue CGRP(-receptor) targeted mAbs after 6 to 12 months is based on expert opinion, as opposed to robust scientific evidence. Current guidelines advise the clinician to assess the success of CGRP(-receptor) targeted mAbs after three months. Based on excellent tolerability data and the absence of scientific data, we propose if no other reasons apply, to stop the use of mAbs when the number of migraine days decreases to four or fewer migraine days per month.

There is a higher likelihood of developing side effects with oral migraine preventatives, and so we suggest stopping these drugs according to the national guidelines if they are well tolerated.

**Conclusion:**

Translational and basic studies are warranted to investigate the long-term effects of a preventive drug after its discontinuation, starting from what is known about the biology of migraine. In addition, observational studies and, eventually, clinical trials focusing on the effect of discontinuation of migraine prophylactic therapies, are essential to substantiate evidence-based recommendations on stopping rules for both oral preventives and CGRP(-receptor) targeted therapies in migraine.

**Graphical Abstract:**

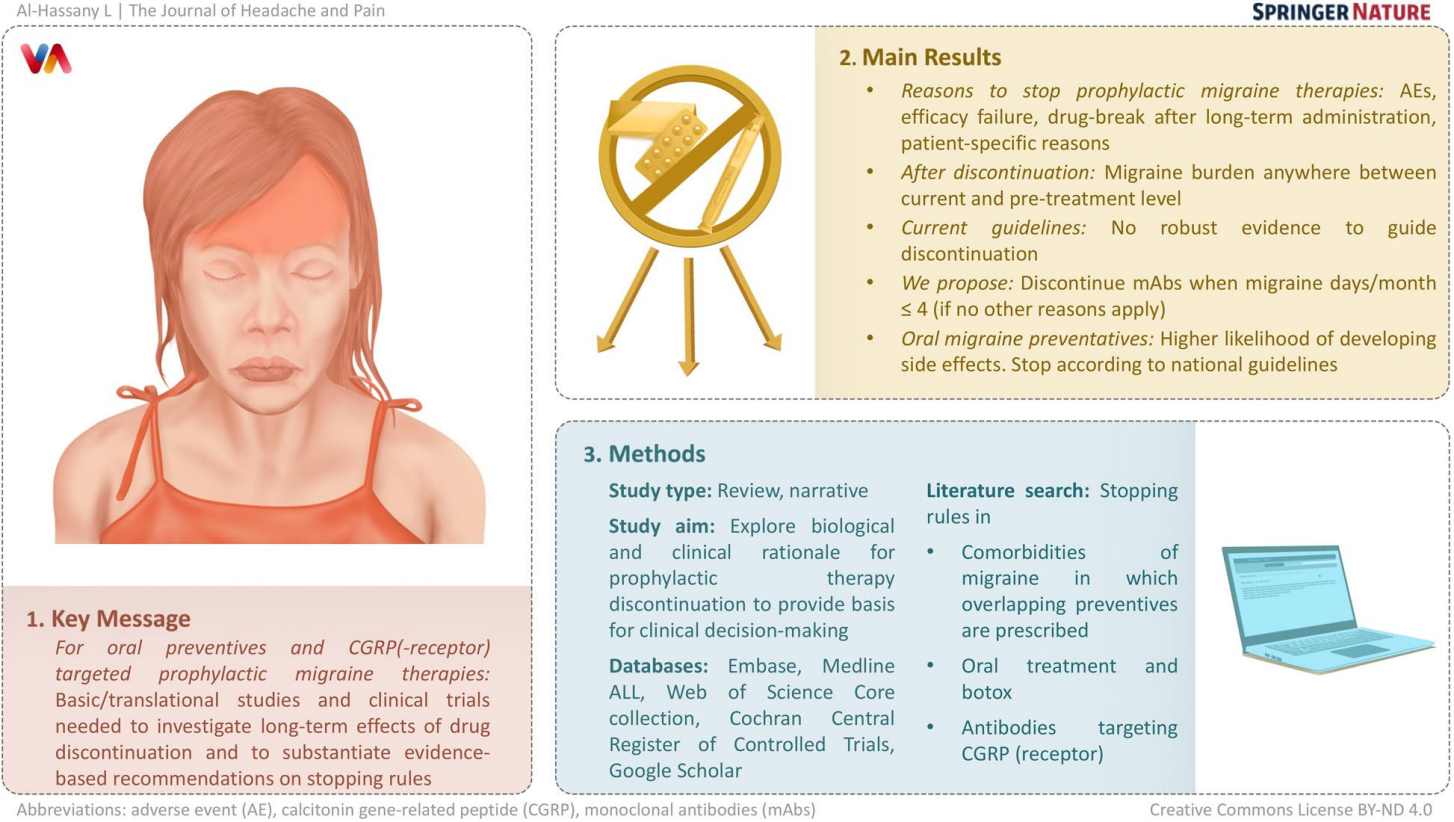

**Supplementary Information:**

The online version contains supplementary material available at 10.1186/s10194-023-01539-8.

## Introduction

Migraine is a primary headache disorder that causes substantial disability and affects over one billion people worldwide [1]. Whilst moderate to severe headache is the cornerstone manifestation of migraine, accompanying symptoms are usually present, such as nausea, photophobia, and phonophobia. Additionally, migraine aura, including sensory, visual, motor, or speech disturbances, may also herald migraine attacks in a subgroup of patients [2]. The wide variety of symptoms encompassing migraine significantly impairs the quality of life [3].

Migraine is ranked as the second most disabling disease globally, and first among women under 50 years old [1, 4]. Accordingly, 11% of the adult population suffer from migraine worldwide, and up to 15% in Europe, with women being three times more affected than men on average [5]. The International Classification of Headache Disorders (ICHD) is constantly evolving, and invaluable in aiding the diagnosis of headache disorders [2]. Migraine constitutes a heterogeneous disease in terms of frequency and severity that can be differentiated as episodic migraine (EM) or chronic migraine (CM). CM is defined as a headache occurring on 15 or more days per month over more than three months, with at least eight days showing migrainous features [2, 6].

Over recent years, there has been a surge in the development of distinctive acute and prophylactic migraine drugs, namely those targeting calcitonin gene-related peptide (CGRP), a nociceptive vasodilating neuropeptide, or its receptor [7]. During a migraine attack, CGRP is released by trigeminal afferents [8] within the trigeminovascular system, and it is thought that sensitization of this system, alongside hyperexcitability of the cerebral cortex, underlies the pathophysiology of migraine [9].

The management of migraine typically consists of acute and prophylactic therapeutics. Preventative medications would be particularly beneficial for patients with frequent and/or severe attacks that affect their quality of life [10]. Various oral medication classes are utilized, including anti-seizure medications (ASM) [11], tricyclic antidepressants, beta-blockers, flunarizine, and angiotensin-II receptor antagonists [12]. Alternative therapies include onabotulinumtoxinA [13] and greater occipital nerve block [14]. Since 2018, the U.S. Food and Drug Administration (FDA) and European Medicines Agency (EMA) approved CGRP mAbs for migraine prevention, including erenumab [15], fremanezumab [16], galcanezumab [17], and lately eptinezumab for migraine prophylaxis in adults [18, 19]. New small-molecule CGRP receptor-targeting medications have also been developed for both abortive and preventative treatment, called gepants, including ubrogepant [20], rimegepant [21], and atogepant [22].

Although leading headache societies provided guidelines and consensus for beginning and escalating migraine prophylactic therapies, robust evidence to guide therapy discontinuation is currently lacking. Our review builds upon hypotheses that have emerged since the introduction of the new CGRP(-receptor) antibodies, namely whether or not the use of these therapies leads to a modification of the chronically evolving disease migraine [23]. Indeed, such disease-modifying characteristics might either be considered as a clinical interference in the natural course of migraine, i.e. reduction of pain parameters and disability, or simply as a deceleration towards chronicity complicated by acute drug abuse. To answer such questions, this review comprehensively explores the biological rationale and clinical studies on migraine preventive therapy discontinuation to better inform clinical management decisions.

## Methods

This is a narrative review of the rationale and current guidelines on stopping migraine prophylaxis. The manuscript was divided into three main topics and, therefore, three different literature main search strategies, namely: i) stopping rules in comorbidities of migraine, namely depression and epilepsy, in which overlapping pharmacological treatments are used; ii) stopping rules of oral treatment and onabotulinumtoxinA (botox), therapies which are traditionally used for migraine prophylaxis; iii) stopping rules of new drugs that are specifically developed for the preventive treatment of migraine, namely CGRP(-receptor) targeted mAbs. While we did not adhere to a systematic approach to screen and include articles on this topic, we performed a systematic search using at least the keywords: “stop*”, “cessation”, “termination”, “rule*”, “guidelines”, “migraine”, “preventive*”, and “prophyla*” to retrieve evidence-based literature, including conference abstracts, covering these topics. References of included articles were screened as well for relevance. Only studies performed in humans were included, while non-English articles were excluded. The following databases were used: *Embase*, *Medline ALL*, W*eb of Science Core Collection*, *Cochrane Central Register of Controlled Trials*, and an additional search engine, namely *Google Scholar* (top 100 ranked). The three search strategies were performed on the 15^th^ and 16^th^ of September 2022, without restrictions in time periods, and details of all search strategies can be found in the Supplementary Materials.

## Results

### Stopping rules in depression and epilepsy therapy

Because there is little pathophysiological explanation for the cessation of migraine prophylactic drugs, we looked at guidance from diseases which can be comorbid with migraine. The most prominent comorbid disorders include depression and anxiety disorders [24]. Because of shared therapeutic medicines, we also looked into epilepsy treatment.

Similar to migraine, the clinical course of depression and epilepsy is variable and the possibility of remission and recovery requires regular reevaluation of the indication of treatment continuation [25, 26].

#### Depression

The administration of an antidepressant usually covers the length of 6 to 12 months in most guidelines. Maintaining successful therapy is recommended due to the increased risk of relapse and recurrent depression. The duration of successful treatment is, similar to migraine prophylaxis, mostly based on consensus rather than evidence [27].

The right time to stop successful therapy with an antidepressant should be chosen on a patient-focused basis. In the decision-making process, the patient's current mood, previous antidepressants, suicide attempts, as well as current risk factors for relapse should be considered [28]. Kendrick et al. assisted patients from primary care practices in discontinuing antidepressant therapy within a controlled randomized study [29]. This study highlighted that fear is an important motive when patients are considering discontinuation of antidepressants; and that clinicians should be aware of their patients’ concerns and expectations.

Similarly, the risk and severity of potential side effects of antidepressants, which occur mostly in elderly, must be factored into further indication for long-term therapy [30]. Severe comorbidities and pain disorders are associated with poorer response to antidepressants and should prompt evaluation by switching or possibly stopping therapy [31]. Selective serotonin reuptake inhibitors (SSRI) are known to decrease platelet aggregation and pose a greater risk of gastrointestinal bleeding, mainly when taken with aspirin and non-steroidal anti-inflammatory drugs [32]. Additionally, there is an increased risk of cardiovascular events, notably myocardial infarctions during therapy with tricyclic antidepressants, which should be stopped or changed in high-risk patients [30]. Other indications to stop an antidepressant may include pregnancy and an inability to take oral medications [28].

Stopping antidepressant therapy is often associated with withdrawal symptoms, such as dizziness, nausea, headache, sleep disturbance, and mood swings. These symptoms can resemble the original symptoms of depression potentially leading to a higher risk of not coping with drug discontinuation [33]. Tapering off antidepressants is intended to give the body time to adapt [34]. Generally, a tapering period of six to eight weeks is recommended for six to eight months of acute therapy, and a tapering period of three to six months is advised for longer maintenance therapy [28]. The shorter the half-life of an antidepressant, the more likely it is that discontinuation symptoms will occur. Therapy duration of at least four to eight weeks increases the risk of withdrawal symptoms upon discontinuation of the drug. Longer maintenance therapy does not appear to further increase the risk of discontinuation symptoms [35].

#### Epilepsy

For the pharmacological treatment of epilepsy, 26 FDA-approved medications are available, and evidence and recommendations for the initiation, efficacy and tolerability of anti-seizure medication (ASM) exist [36].

While numerous studies have addressed the question of stopping ASM in children, respective studies in adults are scarce and recommendations are based on limited evidence [37–40]. To date, the only double-blind randomized controlled study was performed in adult patients on monotherapy, who had been seizure-free for at least two years [41]. The difference in seizure recurrence between patients who continued and discontinued ASM is not significant, whilst neuropsychological tests showed a significant improvement in several domains after withdrawal. The Medical Research Council study included 1013 pediatric and adult patients on polytherapy [42–44]. After two years, the rate of recurrent seizures was significantly higher after ASM withdrawal (41% vs 22%). However, subgroup analyses revealed that the relative risk of seizure recurrence decreased with the length of seizure remission before ASM withdrawal. Consistently, Wang et al. compared the risk of seizure recurrence according to the length of remission before ASM withdrawal [45]. While the recurrence risk was significantly elevated after a remission phase of two years, there was no difference in the risk for patients who had been seizure-free for five or more years of remission.

Based on these studies, the American Academy of Neurology [39], National Institute for Health and Care Excellence (NICE; United Kingdom) [46], and the Italian League Against Epilepsy [38] recommend withdrawal of ASM after a minimum of two years of complete remission. Studies comparing fast to slow withdrawal in children did not reveal evidence for a higher risk of seizure recurrence in one of the groups [37].

In the individual patient, risk factors for seizure recurrence such as younger age at onset of epilepsy, a shorter period of remission, longer duration of active epilepsy, high seizure frequency before seizure remission, ASM polytherapy, and results of the electroencephalogram might be taken into consideration, but do not preclude ASM withdrawal [47–51]. Some of these, and additional factors are included in the online Epilepsy Prediction Tool, designed to estimate the two- and five-year seizure recurrence risk as well as the ten-year chance of seizure freedom based on a study by Lamberink et al. [49, 52].

Furthermore, adverse effects are a leading cause of treatment discontinuation with ASM and occur in up to 96% of patients with varying severity and impairment in the quality of life [53]. However, it has to be taken into consideration that adverse effects of ASM differ in migraine patients compared to epilepsy patients, with stronger adverse effects in migraine patients at the same doses [54]. In contrast to stopping migraine prophylaxis, the withdrawal of ASM requires measures of precaution which are potentially constraining for the patient. More precisely, patients are advised not to drive during withdrawal and for three to six months thereafter, depending on national guidelines [55].

### Stopping rules and their rationale for oral migraine preventatives and onabotulinumtoxinA

Reasons underlying decisions to discontinue migraine pharmacological preventive treatments can be broadly categorized into: (i) adverse events; (ii) efficacy failure; (iii) an attempt to discontinue treatment, despite clinical efficacy, after long-term administration to limit drug exposure; and (iv) patient-specific reasons. Several guidelines and recommendations exist containing both positive and negative stopping rules (i.e., stopping a treatment that is effective or ineffective, respectively) (Table 1).

**Table 1:** Recommendations by various institutes and headache federations. *Abbreviations: CM* = *chronic migraine; EM* = *episodic migraine, MHD = monthly headache days*

Institute or federation	Positive stopping rules	Negative stopping rules	Treatment
National Institute for Health and Care Excellence [56]	Discontinue treatment when a patient has regressed from CM to EM over three consecutive months	Discontinue treatment when a patient does not achieve a reduction of 30% or more in MHDs after two treatment cycles	OnabotulinumtoxinA
European Headache Federation expert consensus [57]	Stop treatment if a patient reaches a reduction to fewer than 10 headache days per month for three consecutive months. Re-evaluate after four to five months	Stop treatment if a patient does not respond during the first two to three treatment cycles	OnabotulinumtoxinA
American College of Physicians and American Academy of Family Physicians [58]	After a period of stability, clinicians are advised to consider tapering or discontinuing treatment, taking into account the patient’s wishes and expectations		All preventatives
British Association for the Study of Headache [59]	Consider gradual withdrawal after 6 to 12 months of effective prophylactic treatment		All preventatives
Canadian Headache Society [60]	After 6 to 12 months of successful prophylactic therapy, consider tapering and discontinuing the prophylactic medication	A prophylactic medication should be administered for at least two months at the maximum tolerated, target, or optimal dose before being considered ineffective	All preventatives

Firstly, drug-related adverse events should promptly result in a shared decision by the physician and the patient on whether to continue or discontinue treatment, considering the broad spectrum of medication classes available for migraine prophylaxis [61]. Some side effects are mainly experienced at the initiation of therapy and may be prevented with a slow up-titration of oral medications; others are dose specific and may be solved by reducing the dosage to levels still able to maintain a sufficient therapeutic effect. Lastly, there are particularly drug-sensitive patients who may experience serious adverse events even with low doses (e.g. cerebellar syndrome after topiramate exposure), warranting a rapid therapy discontinuation.

Secondly, ineffective therapy should also lead to treatment discontinuation. Yet, suboptimal dosage, compliance issues, and inadequate treatment duration must be verified before defining treatment efficacy failure. Multiple efficacy outcome measures might be used to evaluate treatment responses, including migraine-related disability scores (e.g., Migraine Disability Assessment Test (MIDAS)), pain intensity scores, and headache frequency assessed through specific diaries. Nonetheless, 30–50% reduction of monthly headache days (MHDs) is usually considered the most reliable single-efficacy outcome [57, 62, 63]. Negative stopping rules suggest that efficacy outcomes for oral drugs and onabotulinumtoxinA should be assessed after at least two to three months and six to nine months, respectively. Only then, if no clinical improvement is observed, a migraine treatment could be considered ineffective [57, 62].

The third reason underlying therapy discontinuation is the most poorly investigated. The purpose of discontinuing an effective and well-tolerated preventive treatment is to reduce the risks related to unnecessary drug exposure. Patients with 10 to 14 MHDs carry a greater risk of progressing to CM [64, 65]; therefore, 10 MHDs is usually considered the maximum tolerated threshold to safely attempt preventive medication discontinuation. Most recommendations suggest continuing an effective treatment for at least six months before proposing slow drug tapering and eventually suspension, yet evidence to support this practice is lacking [66, 67]. Specifically for onabotulinumtoxinA, it has been suggested or is mandatory according to regulators to stop the treatment in patients who convert from CM to EM or in patients who experience fewer than 10 MHDs for at least three months [57, 68]. Flunarizine, a calcium-channel blocker, should be stopped after six months and only restarted if the patient’s condition relapses. In addition, it is recommended to stop after two months of treatment if no significant improvement is seen [69].

The fourth reason for discontinuing prophylactic treatment comprises patient-specific reasons. These include pregnancy, lactation, financial issues, or patient wishes. Most treatments are contraindicated or not recommended during pregnancy or lactation since clinical trials usually exclude pregnant or breastfeeding women, limiting the generalizability of results in these subgroups of patients [70, 71]. Fortunately, pregnancy and lactation usually have a protective role on migraine burden, therefore, oftentimes, women do not need any pharmacological preventive therapy during this period [72, 73]. Yet, this improvement is more consistently observed in patients with migraine without aura [72, 73]. Moreover, it should be highlighted that the migraine burden fluctuates during the lifetime, hence, there could be periods in a person's life when prophylaxis is no longer needed (e.g. migraine improvement in a subgroup of patients after menopause) [74]. Another reason to stop preventative therapies can occur in some countries when patients do not have the financial means to afford the treatment [75].

When the migraine burden worsens following discontinuation of effective prophylactic treatment, dosage reduction of oral preventatives to the lower effective level could also be considered in order to limit drug exposure [76].

### Results after stopping oral prophylaxis in migraine

Even though positive stopping rules have been proposed by clinical guidelines, most of the studies investigating oral migraine prophylaxis have historically been designed to reveal efficacy outcomes rather than optimal duration or long-lasting effects after discontinuation. Hence, robust evidence to guide clinical practice is currently lacking [76]. Theoretically, following discontinuation, migraine burden may return to the pre-treatment level, remain unchanged, or, more likely, lie somewhere in between.

In the randomized, double-blind study of Diener et al., migraine patients were initially treated with topiramate in a 26-week open-label phase [77]. Participants who adhered to the study protocol until the end of this period entered the 26-week double-blind phase. In this phase, patients were randomly allocated to two groups: a) topiramate continuation and b) topiramate discontinuation with a daily placebo intake. The effect of discontinuation of treatment was investigated. In the placebo discontinuation group, mean monthly migraine days (MMDs) increased by 1.19 days vs 0.10 increase for the topiramate group from the last four weeks of the open-label phase to the last four weeks of the double-blind phase. Additionally, a significant worsening in the treatment discontinuation group compared with the continuation group was observed for headache severity and acute medication intake. Also, the MIDAS score increased by six points in the last four weeks of the double-blind phase compared to the last four weeks of the open-label phase in the placebo group, indicative of a deterioration of quality of life [77]. The relapse after topiramate discontinuation in this study was unaffected by patient characteristics (such as sex, age, and parameters of quality of life) or baseline migraine frequency [78]. Of note, the number of MMDs one month after the treatment discontinuation was significantly increased in patients with low Headache Impact Test-6 (HIT-6) scores who received placebo, but not in patients who received topiramate. Patients in the placebo group were also more likely to relapse if they had reported anxiety or if they had used acute medication during the open-label phase [78]. Pascual et al. observed in another study that discontinuation of topiramate after six months of effective therapy led to a persistent benefit in terms of the number of days with migraine headache in half of the cohort (*n* = 40), while the other half needed to restart preventive medication [79].

Several small studies reinforce these findings in other oral migraine preventive treatments. Flunarizine demonstrated a long-term duration of therapeutic activity after three to six months of effective treatment lasting at least six months, suggesting that this drug may be administered in repeated short treatment cycles to prevent potential long-term side effects [80–83]. Beta-blockers have shown a positive effect after discontinuation following six months of effective treatment persisting for up to six months [84]. Only 25% of patients on amitriptyline and sodium valproate for a duration of six months, with a greater than 50% improvement in the global rating scale and frequency of headache, had a stable remission for one year after treatment discontinuation [84]. Coria et al. investigated discontinuing sodium valproate after three months, and in the third month of follow-up, mean Migraine Assessment Scale (MAS) scores were significantly lower than baseline but significantly higher than at the end of treatment [85]. Rothrock et al. observed a “carry-over effect” (reduction of headache frequency to five days per month) with divalproex sodium in 60% of patients in the first month after cessation, and in 40% of patients, the effect remained for two months or more [86].

Woeber et al. observed in a small study that the reinitiation of a preventative medication class after discontinuation may not be as effective as after the first administration [83]. In line, Raudino et al. mentioned that renewing the prevention with the same medication was ineffective in a group of patients [87]. These data reinforce that identifying baseline predictive factors of sustained response after discontinuation would be pivotal to safely withdrawing an effective preventive treatment.

Several baseline negative prognostic factors that reflect migraine burden and may predict relapse after discontinuation have been identified. These include both clinical (female sex, analgesic overuse, prior treatment failure, ≥ 10 baseline MHDs) and radiological (functional brain changes within the pain matrix) features [76, 83, 88]. Bhoi et al. revealed that the most important predictors of maintained remission after drug discontinuation were improvement at three months after starting prophylactic treatment and a lower prevalence of precipitating factors, namely change in weather, stress, and fasting – triggers that might have been avoided by patients with maintained remission [84].

Taken together, these studies suggest a sustained partial benefit following preventive medications discontinuation for some patients. Nonetheless, consistent studies are present only for topiramate discontinuation, whereas larger randomized, double-blind research studies investigating the discontinuation of other oral common migraine preventive treatments are lacking.

### Stopping rules of CGRP(-receptor) targeted monoclonal antibodies in chronic and episodic migraine

Recommendations on discontinuing CGRP(-receptor) targeted mAbs as a prophylactic treatment of migraine have evolved over the last few years as high-quality data on their safety and efficacy has been collected, both from randomized controlled trials and real-world observations. The recently updated EHF guidelines [7] refer directly to the problem of adequate timing for the first efficacy assessment and possible decision of discontinuing CGRP(-receptor) targeted mAbs.

This review focuses on both negative and positive stopping rules and therefore we do not elaborate on the evaluation period or instruments to determine effective CGRP(-receptor) targeted mAb treatment. When deciding on the “right” duration of prophylactic treatment, there is still an insufficient number of trials that look at determining the optimal timespan for CGRP(-receptor) targeted mAb administration. Additionally, minimal information is available on a possible prolonged prophylactic effect that persists regardless of drug discontinuation. The long half-life of the mAbs [89] and data showing that their efficacy can persist even after five years of treatment [90] support the potential stable benefit from mAbs, but the duration of treatment needed to exert a long-term change that can be sustained after discontinuation is not known yet. Basic information on registered CGRP mAbs is summarized in Table 2.

**Table 2:** General and pharmacokinetic characteristics of CGRP(-receptor) targeted mAbs [89]. *Abbreviations: CGRP* = *calcitonin gene-related peptide; mAbs* = *monoclonal antibodies*

	**Molecular target**	**Indications**	**Administration route**	**Recommended dose and frequency**	**Half-life**
Erenumab	CGRP receptor	Preventive treatment	subcutaneous	70 or 140 mg, monthly	28 days
Fremanezumab	CGRP	Preventive treatment	subcutaneous	225 mg (monthly), 675 mg (quarterly)	31 days
Galcanezumab	CGRP	Preventive treatment	subcutaneous	120 mg (first dose 240 mg), monthly	27 days
Eptinezumab	CGRP	Preventive treatment	intravenous	100 or 300 mg, quarterly	27 days

Another issue that may require additional research is the “wearing off” phenomenon, defined as worsening of the disease control before the next planned dose, which improves after the administration of the drug. Although this effect has been reported by patients treated with erenumab [91], it was not confirmed in clinical trials [92–94].

According to the EHF consensus [7], in both EM and CM, a pause in effective treatment may be considered after 12 to 18 months, and the preventative should be resumed if the frequency of migraine attacks increases. Despite the lack of scientific evidence, experts do not rule out switching from one mAb to another in cases of treatment failure. In this situation, it seems preferable to change to a therapy with a different target (e.g., binding to the CGRP receptor rather than the ligand and vice versa). The above-mentioned principles are also confirmed in other guidelines [95–100]. Therefore, it is important to jointly establish therapeutic goals and possible treatment limitations and to use additional assessment tools, such as quality of life questionnaires during the evaluation.

Particular caution is recommended if a patient no longer meets the reimbursement criteria for prophylactic treatment, but is assessed with a high risk of recurrence, especially in patients with severe, chronic migraine or multiple ineffective attempts of preventive therapy in the past. The stopping rules of CGRP mAbs vary amongst different countries, mainly due to the limitations of reimbursement programs for the use of these drugs. In most guidelines available in English [96–99], the time of the first evaluation is set at three months, after which, if the efficacy criteria are met, treatment may be continued for 6 to 9 [96] or 6 to 12 months [97, 98], until the next evaluation. In the majority of publications, treatment can be considered successful when the reduction of MHDs reaches a minimum of 50%, although in French Headache Society guidelines, the effectiveness threshold for CM was set at a 30% reduction in MMDs. There is agreement among expert organizations as to the need for further research, especially in the context of reinitiation of therapy, and the rationale of switching antibodies in such clinical situations. The recommendations of different organizations are presented in Table 3.

**Table 3:** Criteria for assessing the effectiveness of CGRP(-receptor) targeted mAbs and recommendations for stopping the treatment according to different organisations. *Abbreviations: CGRP* = *calcitonin gene-related peptide; MHD* = *monthly headache days; MIDAS* = *Migraine Disability Assessment Test; MPFID* = *Migraine Physical Function Impact Diary; HIT-6* = *Headache Impact Test- 6*

Institute or Federation	Criteria of effectiveness	Stopping rules
European Headache Federation (EHF) [7]		• Efficacy should be assessed after at least three months of treatment • In EM or CM, a pause in the treatment should be considered after 12 to18 months • Treatment should be continued as long as needed • Restart the treatment if migraine worsens after withdrawal
American Headache Society (AHS) [95]	• Reduction in mean MHDs or headache days of at least moderate severity of at least 50% compared to baseline (documented) • A significant improvement in ANY of the following: MIDAS - Reduction of ≥ 5 points when baseline score is 11 to 20 - Reduction of ≥ 30% when baseline score is > 20 MPFID - Reduction of ≥ 5 points HIT-6 - Reduction of ≥ 5 points	• Efficacy should be assessed after at least three months of treatment for drugs administered monthly and at least six months for drugs administered quarterly • Treatment should be continued only if criteria for effectiveness are met
Germany Society of Neurology and German Migraine and Headache Society [96]	• Reduction in the mean MHDs by ≥ 50% compared to baseline for at least three months (documented) or • Significant improvement in the following: - 30% reduction in MIDAS when baseline score is above 20 - Reduction of ≥ 5 points in HIT-6	• Efficacy should be assessed after at least three months of treatment • Treatment should be continued only if criteria for effectiveness are met • Termination of the therapy should be again considered after six to nine months
French Headache Society [97]	• Reduction of MHDs by: - 50% in EM - 30% in CM • Reduction of acute treatments, intensity and duration of attacks • Improvement of quality of life	• Efficacy should be assessed during the third month of treatment (weeks 8 to 12) • Termination of treatment should be considered due to insufficient efficacy and/or tolerability • Treatment should be continued for 6 to 12 months, then decreased slowly before considering termination • Treatment should be restarted if the frequency of attacks increases again during decrease or after cessation
Danish Headache Society [98]	• Reduction of ≥ 50% in the frequency or severity of migraine • No bothersome side effects	• Efficacy should be assessed after at least two to three months of treatment • Treatment should be terminated if it is not tolerated due to side effect • Treatment should be assessed for discontinuation every 6 to 12 months
Polish Headache Society, the Headache Section of the Polish Neurological Society and the Polish Pain Society [99, 100]	At least one of the following: • Reduction in MHDs/MMDs of 50% compared to baseline (documented) • Improvement in MIDAS score of ≥ 5 points, when a baseline score is 11 to 20 • Reduction in MIDAS score of 30% when a baseline score is close to 20 or a functional improvement in other scores (e.g. MPFID, HIT-6), or improvement documented in the patient’s diary	• Efficacy should be assessed after approximately six months • Treatment should be discontinued if the frequency of attacks remains stable, with a gradual dose reduction • In case of a recurrence of frequent migraine attacks, dose escalation and the continuation of therapy for the next six to nine months are recommended

### The course of migraine after CGRP(-receptor) targeted monoclonal antibody cessation

The suggestion to discontinue prophylactic therapy with CGRP(-receptor) mAbs after 6 to 12 months of successful treatment is based on expert opinion and agrees with recommendations for the use of oral prophylactic drugs in migraine, as per Table 1 [7, 101]. However, multi-year observations assessing discontinuing prophylactic treatment, i.e. drug holidays are still lacking especially considering that mAbs are the youngest prophylactic drugs in migraine and real-life studies are still needed.

A six-month follow-up analysis of two randomized, double-blind, placebo-controlled trials with galcanezumab (EVOLVE-1 and EVOLVE-2) in patients with EM showed marginal worsening of the disease at the end of the study. Migraine frequency remained significantly lower than before randomization for up to four months after the last drug injection. Three months after termination patients reported only one MMD more than during the last treatment month and remained significantly below baseline levels [102]. Raffaelli et al. revealed that 16 patients with CM receiving erenumab or galcanezumab in the open-label extension phase of two clinical trials showed sustained efficacy for three months after study completion, albeit with a slight increase in the number of MMDs over time – from 12.2 days at four weeks after the end of treatment to 14.2 at 12 weeks [103]. Of note, these patients were in a clinical trial setting which could influence the outcome.

Unfortunately, data on the course of migraine after mAb treatment on larger groups of patients is sparse and limited mainly to erenumab, which is probably due to the fact that it was the first mAb available in the world [104, 105]. More than half of the patients experienced early disease worsening, while the remaining patients maintained their good response status in the first four weeks after treatment [104]. Most of the published real-world studies show an increase in MMDs after mAb treatment termination. Gantenbein et al. demonstrated in a retrospective study that 25 of 28 patients showed an increase in MMD in the third month after discontinuation of erenumab [105]. In line with this, a study evaluating the course of migraine after discontinuation of mAb prophylaxis with erenumab, galcanezumab, and fremanezumab in five to eight and 13 to 16 weeks after the last treatment showed that the cessation of prophylaxis was associated with a significant increase in MMD. After four months, the majority of patients were back to baseline migraine frequency prior to the start of prophylaxis with mAbs. The responder rates decreased significantly with only 22.0% of patients having a ≥ 30% reduction in MMD after three months of medication pause vs baseline, and only 10.2% had a reduction of ≥ 50%. Acute medication intake increased in parallel to the increase in MMD [106]. In the entire study, only 10% of patients had a sustained response rate of ≥ 50% in the fourth month after discontinuation of mAbs, and only 8% opted to continue drug vacations beyond week 16. Patients previously on erenumab had a faster deterioration than the other CGRP mAbs, potentially due to its shorter elimination half-life, which is about 21 days. Galcanezumab and fremanezumab have longer bioavailability with half-lives of 27 and 30 days, respectively [107, 108]. In addition, the different mechanisms of action of these mAbs at the level of CGRP or its receptor likely affect their efficacy.

In contrast to prophylaxis with oral medications, issues of tolerability and side effects play a minor role with mAbs. Indeed, it was shown that adverse events did not increase over time and remained similar to what was observed in the placebo group. Yet, as the long-term effects of blockade of the vasodilatory neuropeptide CGRP (or its receptor) – serving as a rescue molecule under ischemic conditions – are not known yet, further studies are warranted to evaluate potential (cardiovascular) side effects of mAbs [109].

Some patients, after discontinuation of CGRP mAbs, will want to resume this medication again. There are few studies looking at the effect on migraine when CGRP mAbs are reintroduced. Raffaelli et al. [110] conducted a longitudinal cohort study (*n* = 39) looking at MMD following reinitiation of a CGRP mAb after a three-month treatment break, including erenumab, galcanezumab, and fremanezumab. A total of 75% of patients responded favourably to the same antibody they had prior to treatment pause while 25% did not. Responders also had a significant improvement in their HIT-6 score. The cause for non-response remains to be determined.

De Matteis et al. [104] studied the effect of treatment resumption with erenumab in 10 patients who were reinitiating erenumab after a minimal pause of four weeks. Eight out of ten patients had a ≥ 50% reduction in MMD during follow-up. This cohort also had a significant reduction in acute medication days compared to baseline.

A prospective cohort study in Italy assessed CM patients treated with erenumab or galcanezumab for 12 months (total *n* = 44) [111]. Subsequently, they discontinued the mAb for three months and then restarted for one month with the same mAb (*n* = 32). Treatment resumption was allowed after one month for those patients who had MMD ≥ 8 and MIDAS score ≥ 11. During the first month after resumption, there was a significant reduction in MMD (− 5.5 ± 8.0) compared with month three of discontinuation. Additionally, there was a significant reduction in the number of acute medication days and HIT-6 score compared with month three of discontinuation. The study by Gantenbein et al. [105] (*n* = 52) looked at patients with either CM (*n* = 21) or EM (*n* = 26) who had received 12 months of treatment with CGRP mAbs (98% erenumab). Of this cohort, 45 patients discontinued CGRP mAbs and of these, 40 patients restarted treatment after 13 ± 3 weeks (range 8–20). Again, MMD following treatment resumption were lower as compared with baseline: 14 ± 7 (EM) and 20 ± 5 (CM), but not as compared with month 12 of treatment: 5 ± 4 (EM) and 5 ± 4 (CM).

Results of these studies suggest that prophylactic medication does not result in long-term changes in neuronal networks most likely because only a very small percentage of these drugs cross the blood–brain barrier [112]. Ziegeler et al. used functional MRI to suggest that erenumab may exert additional modulatory effects on the central processing of trigeminal nociceptive input [112]. Discontinuation of treatment (drug holiday) may be useful to observe the natural improvement of the disease and to evaluate it periodically [7, 106].

### Do underlying mechanisms provide evidence for a treatment termination?

Clear-cut evidence on potential (pathophysiological) mechanisms underlying a sustained benefit or disadvantage of prophylactic treatment cessation is lacking. This is probably related to the fact that the working mechanisms of these drugs in migraine are yet unclear. Therefore, most theoretical explanations of potential (dis)advantages rely on hypotheses and effects observed in other chronic disorders. Here, we hypothesize three main (pathophysiological) mechanisms that might be associated with cessation of prophylactic treatment in migraine patients, namely: (i) disease-modifying effects, that include altering episode sensitization or interictal hyperexcitability, and higher thresholds for attacks; (ii) pharmacodynamic tolerance of prophylactic migraine medications; and (iii) persistent advantages from breaking vicious circles in migraine patients that relate to psychosocial stress.

#### Disease-modifying effects

Some hypotheses exist of an altered interictal hyperexcitability, which could predispose cortical spreading depression and is associated with disbalance or instability of the autonomic nervous system [113]. A sustained effect of prophylactic treatment could be due to an improved stability and balance.

One study assessing this hypothesis was performed by Appel and colleagues. Through spectral analysis of beat-to-beat heart rate fluctuations, they observed that migraine patients have a distributed sympathetic to parasympathetic balance and interictal sympathetic instability [114]. Zigelman et al. investigated whether propranolol, a non-selective beta-adrenoceptor antagonist, can alter this instability after cessation of treatment [115]. Patients who discontinued propranolol after several months of treatment showed a markedly improved sympathetic stability. This so-called “carry-over effect” during the treatment-free period was found for up to two to three months [115]. Thus, a potential mechanism of sustained benefit after treatment cessation could be long-term alteration of sympathetic instability, which is a consequence of autonomic disbalance. Further studies are needed to determine whether similar effects can be expected for the selective beta1-blocker metoprolol and other non-selective beta-adrenoceptor antagonists, such as timolol, and to assess whether this is due to a medication class effect of the beta-blockers. Similar effects of a correction of the sympathetic response have been observed after a one-month treatment period with the calcium-channel blocker verapamil, although this study did not include a period of cessation [116]. Notably, verapamil has no proven efficacy in migraine prophylaxis based on double-blind clinical trials. It remains to be demonstrated whether this improvement in the disbalance of the autonomic nervous system also underlies the effectiveness of other preventives, including other calcium-channel blockers such as flunarizine which also has dopaminergic, serotonergic, and histamine receptor antagonistic effects [117], and candesartan that also influences sympathetic effects. Indeed, candesartan is an antagonist of the angiotensin II receptor that increases sympathetic discharge and release of catecholamines [118].

Another underlying mechanism of a disbalance leading to a lowered threshold in migraine patients is sensitization – either central or of the trigeminovascular pathway [119]. If desensitization could be achieved by prophylactic treatment, this could be a potential mechanism for a sustained effect. One of the prophylactic drugs which may interfere with sensitization and a lowered threshold for the occurrence of migraine attacks is topiramate [77, 120]. Topiramate decreases excitatory neurotransmission via negative modulation of AMPA and kainate receptors and inhibits nociceptive neurotransmission in the trigeminovascular system [120]. The substance also increases the cerebral concentration of the inhibitory neurotransmitter γ-aminobutyric acid (GABA) [119]. Long-term effects of topiramate use have been hypothesized to relate to the reversal of neuronal dysfunction. A sustained and long-term effect of topiramate (and other preventive migraine drugs) might relate to reducing the frequency of migraine attacks, increasing the threshold for future attacks (or baseline of hyperexcitability by suppressing it long enough), and preventing progression to CM [77]. Future studies are needed to support or refute this hypothesis.

The ASMs valproate and divalproex sodium, both used by the body as valproic acid, decrease the degradation of GABA, thereby increasing the concentration of available GABA [121]. This allows for hyperpolarization leading to modulation of calcium, sodium, and potassium ion channels, and so membrane stabilization. It is yet unknown which possible pathophysiological mechanisms underlie a sustained response after a termination period of longer than three months. Indeed, although a sustained benefit was reported after a discontinuation period of six months with topiramate, the number of migraine days increased [77].

#### Pharmacodynamic tolerance

Long-term use of preventives could lead to loss of treatment effectiveness due to pharmacodynamic tolerance [122]. Underlying mechanisms could relate to the upregulation of receptors in case of long-term receptor antagonism with e.g. beta-blockers, angiotensin receptor blockers, and erenumab. Therefore, (abruptly) stopping these treatments could hypothetically lead to new and more severe episodes that increase the chance of recurrence [110]. Along this line of arguments, it is possible that long-term use of topiramate could lead to desensitization of GABA receptors or AMPA and kainate receptors. Whether this has consequences during a drug holiday remains to be determined, as no current evidence-based results are known yet.

In addition, anti-drug antibodies (ADA) have been speculated to play a role in the poor response some patients display using CGRP(-receptor) targeted mAbs. Although neutralizing ADAs against the CGRP(-receptor) targeted mAbs have not been reported to play a role in the efficacy, further studies are needed that investigate the role of developing ADAs after treatment cessation or reinitiation [89, 110, 123].

Similarly, therapies with onabotulinumtoxinA may become ineffective due to the formation of antibodies against onabotulinumtoxinA as is rarely seen in dystonic disorders. Cessation of onabotulinumtoxinA in these patients showed a slow decrease in titres over time [124].

#### Psychosocial vicious circles

Another reason underlying a beneficial effect of preventives that exceeds their treatment period, might be the longer-term advantages related to breaking vicious circles of the migraine disease [125]. An improvement in the migraine course and decrease in migraine attack frequency could lead to less psychosocial stress, and be associated with a healthier lifestyle that, in turn, has a positive influence on the migraine patient [126]. On the other hand, the fear of an increased migraine severity or frequency after treatment cessation or during a pain-free period (cephalalgiaphobia) might lead to an increased intake of acute medications, a worsening of migraine frequency, and medication overuse or CM [127]. Therefore, psychological aspects should be taken into account when considering stopping or continuing preventives.

## Discussion

In this narrative review, we provide evidence and the rationale for stopping migraine prophylaxis in case of successful treatment with these drugs. The purpose to stop unspecific oral migraine preventatives is mainly to omit unwanted pharmacological effects (side effects) and to limit (long-term) drug exposure. Traditionally, the armamentarium of prophylactics in migraine consisted of a variety of (classes of) oral drugs with different modes of action, i.e. beta-blockers, calcium-channel blockers, antidepressants, and anticonvulsants, which were not specifically developed for the treatment of migraine and which show a relatively low adherence rate of 20–30% [128]. The approval of specific anti-migraine prophylaxis, e.g. the CGRP(-receptor) targeted mAbs, has marked a revolutionary era in the prophylactic treatment of migraine. In contrast to the traditional oral therapies, their mode of action in migraine has largely been identified. Off-target effects of these CGRP(-receptor) targeted therapies include usually mild gastrointestinal side effects (constipation and nausea) – similar side effects that are also reported for the oral preventatives [129] – and potential (long-term) cardiovascular side effects that are of special relevance in (postmenopausal) women suffering from migraine with aura, considering their elevated cardiovascular risk [130–132]. Further, the potential involvement of non-canonical receptors (i.e. adrenomedullin and amylin receptors) is still to be determined [133]. Yet, especially compared to the traditional oral preventatives, unwanted clinically relevant (short-term) off-target drug-related effects of CGRP(-receptor) targeted antibodies exist, at least to our current knowledge, only to a minor extent while showing a good efficacy and tolerability. Considering these advantageous characteristics the rules and recommendations for a drug holiday or drug termination in patients with successful mAbs prophylaxis devoid of side effects should be reconsidered.

While exact pathophysiological mechanisms underlying stopping rules for prophylactic drugs in migraine have not been unravelled yet, no obvious rationale for stopping rules exists. Current migraine preventive medications do not seem to possess disease-modifying characteristics, although they exert effects in the central and peripheral nervous system and knowledge on potential pharmacodynamic desensitization is lacking. Disease modification would allow short-term prophylaxis with long-term benefits. Clinical trials and larger real-world observations assessing the effects of termination of oral preventatives are limited, and current data do not provide robust evidence for long-term benefits beyond the period of medication intake. In line, data on drugs targeting the CGRP pathway do not indicate long-term changes of the disease. Of note, most data are derived from studies with a maximum treatment duration of 12 months and studies on mAbs with a duration longer than five years are not available yet, considering their relatively recent introduction into the market. This brings limitations to this statement.

The main question is whether the treatment goal of migraine prophylaxis is to gain complete migraine freedom for patients, freedom from migraine-related disability, or only a reduction in migraine attacks before termination of prophylaxis. The latter would only mean control of the disease. Of note, months with complete migraine freedom can be achieved with CGRP(-receptor) targeted therapies, or at least in super responders to mAbs (≥ 75% response) [134]. Yet, complete migraine freedom for more than three months in a row is rare at this stage. In this context, the higher placebo effect of mAbs compared to other (oral) preventative drugs should be considered [135], which – in its turn – might have beneficial effects on the vicious psychosocial circle that could either worsen or improve the course of migraine.

In epilepsy, the aim is to achieve seizure freedom by sometimes combining multiple drugs for a defined period of time. In the therapy of depression, symptom control is the aim in the absence of specific medications and due to the use of substances with multiple effects. Migraine therapy has reached a stage beyond the stage of therapy in depression due to the knowledge of the key neuropeptides (e.g. CGRP, but also pituitary adenylate cyclase-activating polypeptide, neuropeptide Y, and vasoactive intestinal peptide) involved in the onset and progression of migraine, although we do not yet completely understand their (relative) contributions, especially in the context of the heterogeneous manifestations and treatment responses in migraine patients. Yet, directly comparing stopping rules between migraine treatment and the therapy of epilepsy and depression should be done with caution, as depression and epilepsy are usually treated with different drugs and doses compared with migraine, even when migraine is treated with antidepressants or ASM. Furthermore, while epilepsy is a chronic condition with episodic manifestations, just like migraine, depression has a different disease course.

Therefore, while ideally, our treatment goals in migraine prophylaxis should exceed disease control towards attack freedom or freedom from migraine-related disability, this might be unrealistic in all patients, but one should at least aim to come as close to migraine freedom and freedom of migraine-related disability as possible – if the medication is well tolerated. Indeed, oral preventatives show a variety of side effects that range from gastrointestinal symptoms to tachycardia and urinary retention [136], which might contribute to the valid decision not to reach complete migraine freedom.

The indication of migraine prophylaxis depends on national guidelines and can broadly be described as migraine attacks that affect the patients’ quality of life. However, the extent or magnitude of quality of life affection as a reason for migraine prophylaxis has not been determined, at least to our knowledge, and needs to be studied. One could speculate that migraine prophylaxis can be stopped when, after a certain period of time, quality of life – determined by migraine attack frequency or intensity – has significantly improved and when the use of acute medications has decreased. A caveat of this approach is that it does not take into account that migraine as an active disease is still existing and that patient preference should always be taken into account.

**Fig. 1:**
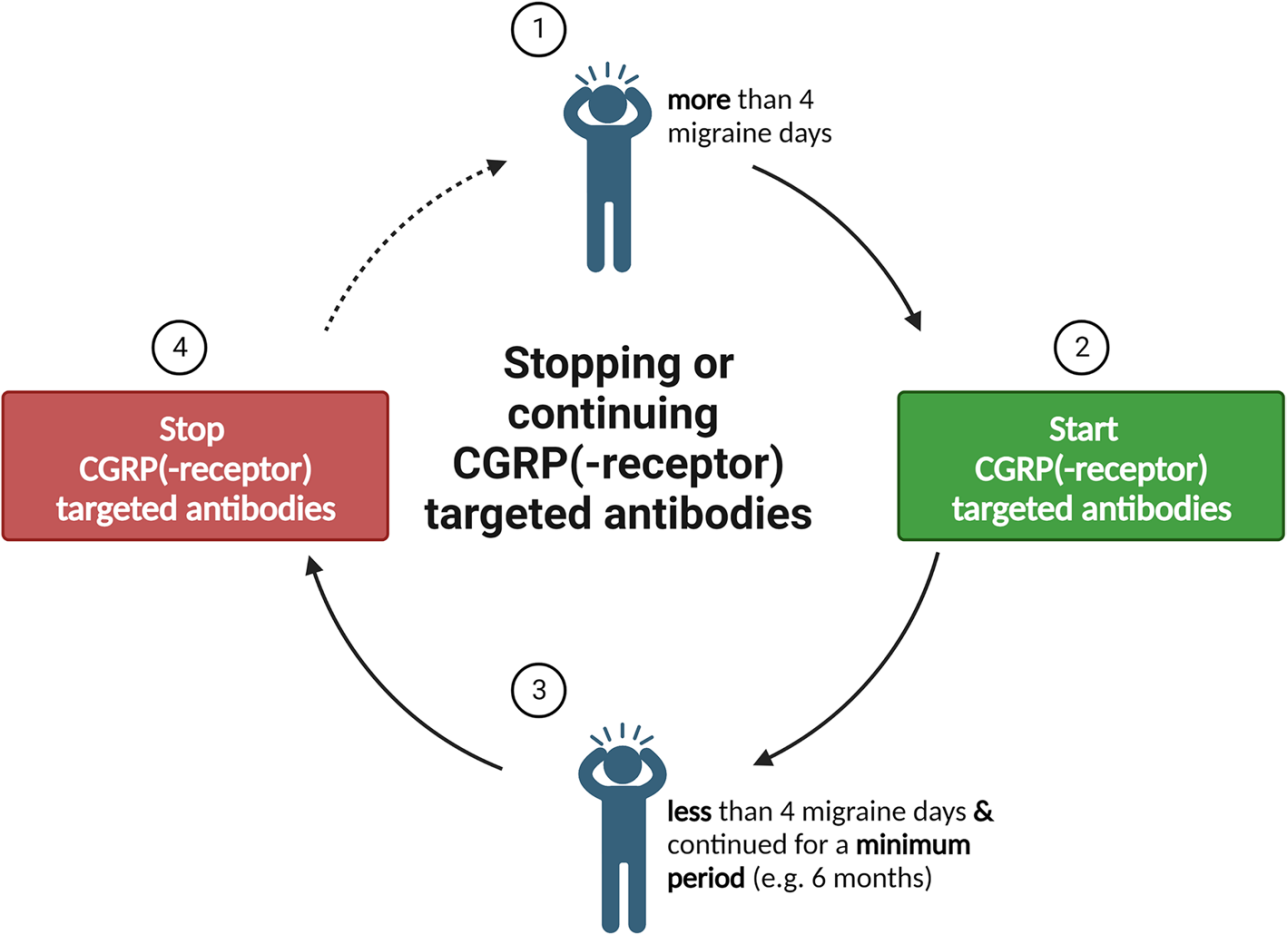
An illustrative tool for stopping or continuing CGRP(-receptor) targeted antibodies. *Abbreviation: CGRP* = *calcitonin gene-related peptide*

Obviously, there is a conflict related to migraine prophylaxis. On one side, a desire for pain freedom and complete disease control exists, which cannot be achieved today based on the efficacy of available medications and the complexity of the disease. On the other hand, disease control or disease improvement does not seem to be sufficient anymore when using specific medications. In order to solve this conflict and to advance the field it is time to consider new goals that should be achieved before stopping prophylaxis. A look into the indication of CGRP(-receptor) targeted mAb therapies may provide further insight. A level of at least four MMDs justifies the use of these drugs by medical authorities in the absence of reimbursement regulations. Therefore, a next step towards stopping prophylaxis with these drugs is the lack of an indication for such therapies which would be less than four MMDs under mAb treatment (Fig. 1). Data show that migraine disability increases with attack frequency [137]. However, a significant reduction of MMDs to approximately four MMDs after a 12-month treatment of monthly erenumab 70 mg in EM patients is still associated with significant migraine-related disability as indicated by a HIT-6 score of 51.7 points. As long as disability exists prophylaxis is indicated.

**Fig. 2:**
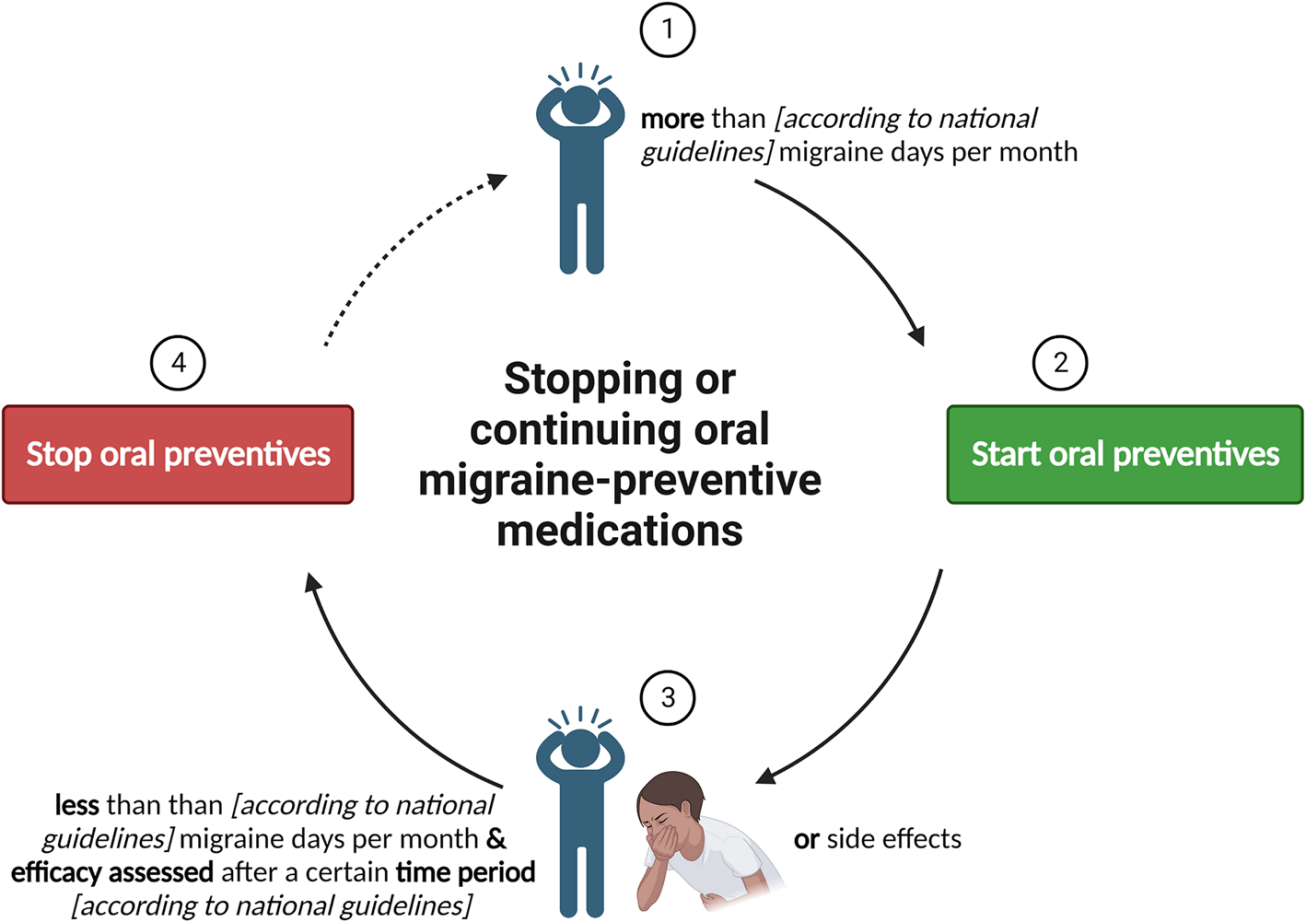
An illustrative tool for stopping or continuing oral migraine preventive medications

There remains controversy over whether, in the case of adverse events, we stop a medication completely, or switch to another medication in the same class, i.e. CGRP(-receptor) targeted mAbs. Patients will additionally have differing levels of tolerability, with some people unwilling to remain on a medication with mild side effects, to others tolerating ‘worse’ side effects when weighed up against the severity of their migraines. Considering the lack of evidence on oral preventives and the higher likelihood of developing side effects, we suggest stopping these drugs according to the national guidelines, but only if they are well tolerated (Fig. 2). Yet, in migraine patients with comorbidities (e.g. depression or epilepsy) or risk factors for migraine progression, caution is advised.

The hypothesis to continue monoclonal antibodies acting on the CGRP pathway for longer periods compared to traditional preventives, is not based on its influences on the course of migraine. Indeed, monoclonal antibodies might not be disease-modifying, and therefore, they do not have clear efficacy advantages over traditional drugs. Yet, instead, they are way more tolerable due to their specific action, supporting their longer treatment period.

For onabotulinumtoxinA, in contrast, stopping rules are longer than those of oral preventive drugs. This is due to its intramuscular administration, which bypasses metabolization via the gastro-intestinal route and avoids interactions with e.g. cytochrome P450 enzymes, in contrast to oral preventives. Further, the duration of action is notably longer and the tolerability of onabotulinumtoxinA is generally better compared to oral drugs, especially considering the side effects of oral prophylactic drugs resulting in a low persistence [138].

Several limitations are associated with this narrative review. Whilst multiple systematic search engines were utilized with keywords, the identification of articles and results was not conducted in a systematic manner. We also did not include non-English articles. Further, the scope of this review did not include acute treatment or non-pharmacological management. Indeed, there is increasing evidence emerging on the use of alternative therapies for chronic pain. In addition, the mentioned studies use a variety of outcome measures that hamper a proper comparison to provide clear-cut evidence and the conduct of a systematic (rather than a narrative) review on stopping preventive therapies. Indeed, the flexible use of MMDs and MHDs provides room for error when utilized interchangeably. This also highlights the importance of headache diaries and the documentation of headache free days in future studies. Through the use of headache diaries, we can reduce recall bias, and more accurately diagnose the headache, along with initiating, monitoring, and discontinuing treatment [139]. Indeed, it should be realized that clinical trials are not necessarily generalizable to the overall population, considering the phenomenon of regression to the mean, particularly during the open-label phases on preventive antimigraine drugs. Patients tend to agree to participate in clinical trials at times when their migraines are particularly bad.

For our included studies, the duration is often six months to one year. This highlights the need for longer studies investigating long-term efficacy and long-term discontinuation effects. As gepants and CGRP(-receptor) targeted mAbs become more widely available to EM and CM patients, more robust clinical trials will need to be conducted – not only looking at efficacy, but also discontinuation effects. Furthermore, more basic studies are needed to better understand the pathophysiological mechanisms that accompany stopping (oral) prophylactic drugs and the neurobiological processes that underlie migraine progression [140]. Ultimately, the identification of (several) objective biomarkers for disease improvement and prognostic (clinical) features of treatment relapse would bring advantages in determining whether and which migraine patients would benefit from discontinuing migraine prophylaxis.

## Conclusion

Current guidelines advise clinicians to assess the success of CGRP(-receptor) targeted mAbs after three months and to continue when the treatment is successful. Based on excellent tolerability data and in the absence of scientific data, we propose if no other reasons apply, to stop the use of mAbs when the number of migraine days decreases to four or less migraine days per month. For oral preventives, we advise adhering to national guidelines, yet to be cautious in case of side effects, risk factors for migraine progression, and the presence of comorbidities. Patients' preferences and psychosocial factors should at all times be taken into account. Clinical trials with a focus on the effect of discontinuation of migraine prophylactic therapies and basic or translational studies to enhance our understanding of underlying mechanisms that accompany discontinuation are warranted.

## Supplementary Information

**Additional file 1.**

## Data Availability

Not applicable.

## Abbreviations

ADA: Anti-drug antibodies
AMPA: α-Amino-3-hydroxy-5-methyl-4-isoxazolepropionic acid
ASM: Anti-seizure medication
CI: Confidence interval
CM: Chronic migraine
CGRP: Calcitonin gene-related peptide
EHF: European Headache Federation
EM: Episodic migraine
EMA: European Medicines Agency
FDA: U.S. Food and Drug Administration
GABA: Gamma-aminobutyric acid
HIT-6: Headache Impact Test
ICHD: International Classification of Headache Disorders
mAb: Monoclonal antibody
MHD: Monthly headache days
MIDAS: Migraine Disability Assessment Test
MMD: Monthly migraine days
MPFID: Migraine Physical Function Impact Diary
NICE: National Institute For Health and Care Excellence
SSRI: Selective serotonin reuptake inhibitor
