# Supplementary material for: The sense of stopping migraine prophylaxis
Source: J Headache Pain. 2023 Feb 16;24(1):9. doi: 10.1186/s10194-023-01539-8 (PMC9933401; doi:10.1186/s10194-023-01539-8)
Supplement: Supplementary file 1 — Additional file 1. [file 10194_2023_1539_MOESM1_ESM.docx]

**SUPPLEMENTARY MATERIALS**

**Search strategy I – Oral preventatives in migraine**

*Performed on the 15^th^ of September 2022*

| **Database searched** | **Platform** | **Years of coverage** | **Records** | **Records after duplicates removed** |
| --- | --- | --- | --- | --- |
| Embase | Embase.com | 1971 - Present | 876 | 822 |
| Medline ALL | Ovid | 1946 - Present | 174 | 27 |
| Web of Science Core Collection* | Web of Knowledge | 1975 - Present | 172 | 23 |
| Cochrane Central Register of Controlled Trials | Wiley | 1992 - Present | 232 | 71 |
| Additional Search Engines: Google Scholar (100 top ranked) | | | 100 | 91 |
| **Total** | | | **1554** | **1034** |

**Science Citation Index Expanded (1975-present) ; Social Sciences Citation Index (1975-present) ; Arts & Humanities Citation Index (1975-present) ; Conference Proceedings Citation Index- Science (1990-present) ; Conference Proceedings Citation Index- Social Science & Humanities (1990-present) ; Emerging Sources Citation Index (2005-present)*

*No other database limits were used than those specified in the search strategies*

**Embase**

(migraine/exp/'prevention' OR (migraine/mj/exp AND ('prevention'/mj OR 'prophylaxis'/mj)) OR (((migraine*) NEAR/3 (prevent* OR prophyla*))):ab,ti,kw OR (migraine* AND (prevent* OR prophyla*)):ti) **AND** ('drug holiday'/de OR 'drug withdrawal'/de OR 'treatment interruption'/de OR (((drug* OR medication* OR rule* OR treatment* OR therap* OR guideline* OR strateg* OR prophyla*) NEAR/3 (withdraw* OR cessation* OR termination* OR stopping OR ceas* OR stop OR discontinu* OR interrupt* OR gap OR gaps OR holiday* OR withhold*))):ab,ti,kw) NOT ([animals]/lim NOT [humans]/lim) NOT ('drug induced headache'/mj OR (overuse-headache* OR drug-induced* OR analges*-induced OR medicat*-overuse* OR analges*-overuse* OR ((drug* OR headache* OR medicat* OR analges*) NEAR/3 (misuse* OR overuse* OR abuse))):ti,kw) AND [ENGLISH]/lim

**Medline**

(exp Migraine Disorders/pc OR (*Migraine Disorders/ AND (prevention and control/ OR Secondary Prevention /)) OR (((migraine*) ADJ3 (prevent* OR prophyla*))).ab,ti,kf. OR (migraine* AND (prevent* OR prophyla*)).ti.) **AND** (Withholding Treatment/ OR Drug Tapering/ OR (((drug* OR medication* OR rule* OR treatment* OR therap* OR guideline* OR strateg* OR prophyla*) ADJ3 (withdraw* OR cessation* OR termination* OR stopping OR ceas* OR stop OR discontinu* OR interrupt* OR gap OR gaps OR holiday* OR withhold*))).ab,ti,kf.) NOT (exp animals/ NOT humans/) NOT ((overuse-headache* OR drug-induced* OR analges*-induced OR medicat*-overuse* OR analges*-overuse* OR ((drug* OR headache* OR medicat* OR analges*) ADJ3 (misuse* OR overuse* OR abuse))).ti,kf) AND english.la.

**Cochrane**

((((migraine*) NEAR/3 (prevent* OR prophyla*))):ab,ti,kw OR (migraine* AND (prevent* OR prophyla*)):ti) **AND** ((((drug* OR medication* OR rule* OR treatment* OR therap* OR guideline* OR strateg* OR prophyla*) NEAR/3 (withdraw* OR cessation* OR termination* OR stopping OR ceas* OR stop OR discontinu* OR interrupt* OR gap OR gaps OR holiday* OR withhold*))):ab,ti,kw) NOT ((overuse NEXT headache* OR drug NEXT induced* OR analges* NEXT induced OR medicat* NEXT overuse* OR analges* NEXT overuse* OR ((drug* OR headache* OR medicat* OR analges*) NEAR/3 (misuse* OR overuse* OR abuse))):ti)

**Web of Science**

(TS=(((migraine*) NEAR/2 (prevent* OR prophyla*))) OR TI=(migraine* AND (prevent* OR prophyla*))) **AND** TS=(((drug* OR medication* OR rule* OR treatment* OR therap* OR guideline* OR strateg* OR prophyla*) NEAR/2 (withdraw* OR cessation* OR termination* OR stopping OR ceas* OR stop OR discontinu* OR interrupt* OR gap OR gaps OR holiday* OR withhold*))) NOT TI=(overuse-headache* OR drug-induced* OR analges*-induced OR medicat*-overuse* OR analges*-overuse* OR ((drug* OR headache* OR medicat* OR analges*) NEAR/2 (misuse* OR overuse* OR abuse))) NOT TS=((animal* OR rat OR rats OR mouse OR mice OR murine OR dog OR dogs OR canine OR cat OR cats OR feline OR rabbit OR cow OR cows OR bovine OR rodent* OR sheep OR ovine OR pig OR swine OR porcine OR veterinar* OR chick* OR zebrafish* OR baboon* OR nonhuman* OR primate* OR cattle* OR goose OR geese OR duck OR macaque* OR avian* OR bird* OR fish*) NOT (human* OR patient* OR women OR woman OR men OR man)) AND LA=(English)

**Google Scholar**

migraine prevention|prophylaxis|prophylactic "stopping|interruption|cessation|holiday|termination|stopping|discontinue|withholding rules|guidelines|strategy"

migraine prevention|prophylaxis|prophylactic 'stopping|interruption|cessation|holiday|termination|stopping|discontinue|withholding rules|guidelines|strategy'

**Search strategy II – CGRP(-receptor) targeted therapies in migraine**

*Performed on the 16^th^ of September 2022*

| **Database searched** | **Platform** | **Years of coverage** | **Records** | **Records after duplicates removed** |
| --- | --- | --- | --- | --- |
| Medline ALL | Ovid | 1946 - Present | 65 | 65 |
| Embase | Embase.com | 1971 - Present | 344 | 230 |
| Web of Science Core Collection* | Web of Knowledge | 1975 - Present | 79 | 11 |
| Cochrane Central Register of Controlled Trials | Wiley | 1992 - Present | 103 | 9 |
| Additional Search Engines: Google Scholar (100 top-ranked) | | | 100 | 76 |
| **Total** | | | **691** | **391** |

**Science Citation Index Expanded (1975-present) ; Social Sciences Citation Index (1975-present) ; Arts & Humanities Citation Index (1975-present) ; Conference Proceedings Citation Index- Science (1990-present) ; Conference Proceedings Citation Index- Social Science & Humanities (1990-present) ; Emerging Sources Citation Index (2005-present)*

*No other database limits were used than those specified in the search strategies*

**Embase**

(migraine/exp OR (migraine* OR antimigrain*):ab,ti,kw) **AND** ('prevention'/exp OR 'prophylaxis'/exp OR (prevent* OR prophyla*):ab,ti,kw) **AND** ('calcitonin gene related peptide'/de OR Atogepant/de OR Rimegepant/de OR Ubrogepant/de OR Zavegepant/de OR Erenumab/de OR Eptinezumab/de OR Fremanezumab/de OR Galcanezumab/de OR (calcitonin-gene-related-peptide* OR CGRP* OR Atogepant* OR Rimegepant* OR Ubrogepant* OR Zavegepant* OR Erenumab* OR Eptinezumab* OR Fremanezumab* OR Galcanezumab* OR vazegepant* OR AGN241689 OR AGN-241689 OR Aimovig OR Ajovy OR ALD403 OR ALD-403 OR AMG334 OR AMG-334 OR BHV3000 OR BHV-3000 OR BHV3500 OR BHV-3500 OR BMS742413 OR BMS-742413 OR BMS927711 OR BMS-927711 OR Emgality OR LY2951742 OR MK1602 OR MK-1602 OR MK8031 OR MK-8031 OR Nurtec-ODT OR TEV48125 OR TEV-48125 OR Ubrelvy OR Vyepti ):ab,ti,kw) **AND** ('drug holiday'/de OR 'drug withdrawal'/de OR 'treatment interruption'/de OR (((drug* OR medication* OR rule* OR treatment* OR therap* OR guideline* OR strateg* OR prophyla*) NEAR/6 (withdrawal* OR cessation* OR termination* OR stopping OR ceas* OR stop OR discontinu* OR interrupt* OR gap OR gaps OR holiday* OR withhold*))):ab,ti,kw) NOT ([animals]/lim NOT [humans]/lim) NOT ('drug induced headache'/mj OR (overuse-headache* OR drug-induced* OR analges*-induced OR medicat*-overuse* OR analges*-overuse* OR ((drug* OR headache* OR medicat* OR analges*) NEAR/3 (misuse* OR overuse* OR abuse))):ti,kw) AND [ENGLISH]/lim

**Medline**

(exp Migraine Disorders/ OR (migraine* OR antimigrain*).ab,ti,kf.) **AND** (pc.fs. OR Secondary Prevention/ OR (prevent* OR prophyla*).ab,ti,kf.) **AND** (Calcitonin Gene-Related Peptide/ OR atogepant.nm. OR rimegepant sulfate.nm OR ubrogepant.nm OR erenumab.nm OR eptinezumab.nm OR fremanezumab.nm OR galcanezumab.nm OR (calcitonin-gene-related-peptide* OR CGRP* OR Atogepant* OR Rimegepant* OR Ubrogepant* OR Zavegepant* OR Erenumab* OR Eptinezumab* OR Fremanezumab* OR Galcanezumab* OR vazegepant* OR AGN241689 OR AGN-241689 OR Aimovig OR Ajovy OR ALD403 OR ALD-403 OR AMG334 OR AMG-334 OR BHV3000 OR BHV-3000 OR BHV3500 OR BHV-3500 OR BMS742413 OR BMS-742413 OR BMS927711 OR BMS-927711 OR Emgality OR LY2951742 OR MK1602 OR MK-1602 OR MK8031 OR MK-8031 OR Nurtec-ODT OR TEV48125 OR TEV-48125 OR Ubrelvy OR Vyepti ).ab,ti,kf.) **AND** (Withholding Treatment/ OR Drug Tapering/ OR (((drug* OR medication* OR rule* OR treatment* OR therap* OR guideline* OR strateg* OR prophyla*) ADJ6 (withdrawal* OR cessation* OR termination* OR stopping OR ceas* OR stop OR discontinu* OR interrupt* OR gap OR gaps OR holiday* OR withhold*))).ab,ti,kf.) NOT (exp animals/ NOT humans/) NOT ((overuse-headache* OR drug-induced* OR analges*-induced OR medicat*-overuse* OR analges*-overuse* OR ((drug* OR headache* OR medicat* OR analges*) ADJ3 (misuse* OR overuse* OR abuse))).ti,kf.) AND english.la.

**Cochrane**

((migraine* OR antimigrain*):ab,ti,kw) **AND** ((prevent* OR prophyla*):ab,ti,kw) **AND** ( (calcitonin NEXT gene NEXT related NEXT peptide* OR CGRP* OR Atogepant* OR Rimegepant* OR Ubrogepant* OR Zavegepant* OR Erenumab* OR Eptinezumab* OR Fremanezumab* OR Galcanezumab* OR vazegepant* OR AGN241689 OR AGN NEXT 241689 OR Aimovig OR Ajovy OR ALD403 OR ALD NEXT 403 OR AMG334 OR AMG NEXT 334 OR BHV3000 OR BHV NEXT 3000 OR BHV3500 OR BHV NEXT 3500 OR BMS742413 OR BMS NEXT 742413 OR BMS927711 OR BMS NEXT 927711 OR Emgality OR LY2951742 OR MK1602 OR MK NEXT 1602 OR MK8031 OR MK NEXT 8031 OR Nurtec NEXT ODT OR TEV48125 OR TEV NEXT 48125 OR Ubrelvy OR Vyepti ):ab,ti,kw) **AND** ((((drug* OR medication* OR rule* OR treatment* OR therap* OR guideline* OR strateg* OR prophyla*) NEAR/6 (withdrawal* OR cessation* OR termination* OR stopping OR ceas* OR stop OR discontinu* OR interrupt* OR gap OR gaps OR holiday* OR withhold*))):ab,ti,kw) NOT ((overuse NEXT headache* OR drug NEXT induced* OR analges* NEXT induced OR medicat* NEXT overuse* OR analges* NEXT overuse* OR ((drug* OR headache* OR medicat* OR analges*) NEAR/3 (misuse* OR overuse* OR abuse))):ti)

**Web of Science**

TS=((((migraine* OR antimigrain*)) **AND** ((prevent* OR prophyla*)) **AND** ( (calcitonin-gene-related-peptide* OR CGRP* OR Atogepant* OR Rimegepant* OR Ubrogepant* OR Zavegepant* OR Erenumab* OR Eptinezumab* OR Fremanezumab* OR Galcanezumab* OR vazegepant* OR AGN241689 OR AGN-241689 OR Aimovig OR Ajovy OR ALD403 OR ALD-403 OR AMG334 OR AMG-334 OR BHV3000 OR BHV-3000 OR BHV3500 OR BHV-3500 OR BMS742413 OR BMS-742413 OR BMS927711 OR BMS-927711 OR Emgality OR LY2951742 OR MK1602 OR MK-1602 OR MK8031 OR MK-8031 OR Nurtec-ODT OR TEV48125 OR TEV-48125 OR Ubrelvy OR Vyepti )) **AND** ((((drug* OR medication* OR rule* OR treatment* OR therap* OR guideline* OR strateg* OR prophyla*) NEAR/5 (withdrawal* OR cessation* OR termination* OR stopping OR ceas* OR stop OR discontinu* OR interrupt* OR gap OR gaps OR holiday* OR withhold*))))) NOT ((animal* OR rat OR rats OR mouse OR mice OR murine OR dog OR dogs OR canine OR cat OR cats OR feline OR rabbit OR cow OR cows OR bovine OR rodent* OR sheep OR ovine OR pig OR swine OR porcine OR veterinar* OR chick* OR zebrafish* OR baboon* OR nonhuman* OR primate* OR cattle* OR goose OR geese OR duck OR macaque* OR avian* OR bird* OR fish*) NOT (human* OR patient* OR women OR woman OR men OR man))) NOT TI=(overuse-headache* OR drug-induced* OR analges*-induced OR medicat*-overuse* OR analges*-overuse* OR ((drug* OR headache* OR medicat* OR analges*) NEAR/2 (misuse* OR overuse* OR abuse))) AND LA=(English)

**Google Scholar**

migraine prevention|prophylaxis|prophylactic CGRP|Atogepant|Rimegepant|Ubrogepant|Zavegepant|Erenumab|Eptinezumab|Fremanezumab|Galcanezumab|vazegepant stopping|interruption|cessation|tapering|termination| discontinue|withholding

**Search strategy III – Stopping rules in depression and epilepsy therapy**

*Performed on the 16^th^ of September 2022*

| **Database searched** | **Platform** | **Years of coverage** | **Records** | **Records after duplicates removed** |
| --- | --- | --- | --- | --- |
| Embase | Embase.com | 1971 - Present | 142 | 140 |
| Medline ALL | Ovid | 1946 - Present | 28 | 8 |
| Web of Science Core Collection* | Web of Knowledge | 1975 - Present | 20 | 6 |
| Cochrane Central Register of Controlled Trials | Wiley | 1992 - Present | 23 | 23 |
| Additional Search Engines: Google Scholar | | | 100 | 100 |
| **Total** | | | **313** | **277** |

**Science Citation Index Expanded (1975-present) ; Social Sciences Citation Index (1975-present) ; Arts & Humanities Citation Index (1975-present) ; Conference Proceedings Citation Index- Science (1990-present) ; Conference Proceedings Citation Index- Social Science & Humanities (1990-present) ; Emerging Sources Citation Index (2005-present)*

*No other database limits were used than those specified in the search strategies*

**Embase**

('anticonvulsive agent'/mj/exp OR 'antidepressant agent'/mj/exp OR (anticonvulsant* OR anti-convulsant* OR anticonvulsiv* OR anti-convulsiv* OR antiepileptic* OR anti-epileptic* OR antidepress* OR anti-depress* OR ((botulin*) NEAR/3 (toxin*)) OR botox OR onabotulinumtoxin* OR incobotulinumtoxin*):ti,kw) **AND** ((('practice guideline'/mj/exp OR 'consensus'/mj/exp OR 'clinical practice'/mj/exp) AND ('drug withdrawal'/de OR 'treatment withdrawal'/de OR 'treatment interruption'/de OR 'drug dose reduction'/de)) OR (((rule* OR guideline* OR guide OR guidance* OR strateg* OR guidance* OR consensus* OR recommendation* OR clinical-practic*) AND (withdraw* OR cessation* OR termination* OR stopping OR ceas* OR stop OR discontinue* OR interrupt* OR holiday* OR withhold* OR dose-reduct* OR tapering*))):ti,kw) NOT ([animals]/lim NOT [humans]/lim) AND [ENGLISH]/lim NOT ('smoking cessation'/mj OR 'smoking'/mj/exp OR (smok* OR tobacco*):ti)

**Medline**

(exp *Anticonvulsants/ OR exp *Antidepressive Agents/ OR (anticonvulsant* OR anti-convulsant* OR anticonvulsiv* OR anti-convulsiv* OR antiepileptic* OR anti-epileptic* OR antidepress* OR anti-depress* OR ((botulin*) ADJ3 (toxin*)) OR botox OR onabotulinumtoxin* OR incobotulinumtoxin*).ti,kf.) **AND** (((Practice Guideline.pt. OR *Practice Guidelines as Topic/ OR Guideline.pt OR *Consensus/ OR *Clinical Decision Rules /) AND (Withholding Treatment/ OR Drug Tapering/)) OR (((rule* OR guideline* OR guide OR guidance* OR strateg* OR guidance* OR consensus* OR recommendation* OR clinical-practic*) AND (withdraw* OR cessation* OR termination* OR stopping OR ceas* OR stop OR discontinue* OR interrupt* OR holiday* OR withhold* OR dose-reduct* OR tapering*))).ti,kf.) NOT (exp animals/ NOT humans/) AND english.la. NOT (*Smoking Cessation/ OR exp *Smoking/ OR (smok* OR tobacco*).ti.)

**Cochrane**

((anticonvulsant* OR anti-convulsant* OR anticonvulsiv* OR anti-convulsiv* OR antiepileptic* OR anti-epileptic* OR antidepress* OR anti-depress* OR ((botulin*) NEAR/3 (toxin*)) OR botox OR onabotulinumtoxin* OR incobotulinumtoxin*):ti,kw) **AND** ((((rule* OR guideline* OR guide OR guidance* OR strateg* OR guidance* OR consensus* OR recommendation* OR clinical-practic*) AND (withdraw* OR cessation* OR termination* OR stopping OR ceas* OR stop OR discontinue* OR interrupt* OR holiday* OR withhold* OR dose-reduct* OR tapering*))):ti,kw) NOT ((smok* OR tobacco*):ti)

**Web of Science**

TI=((anticonvulsant* OR anti-convulsant* OR anticonvulsiv* OR anti-convulsiv* OR antiepileptic* OR anti-epileptic* OR antidepress* OR anti-depress* OR ((botulin*) NEAR/2 (toxin*)) OR botox OR onabotulinumtoxin* OR incobotulinumtoxin*) **AND** ((rule* OR guideline* OR guide OR guidance* OR strateg* OR guidance* OR consensus* OR recommendation* OR clinical-practic*) AND (withdraw* OR cessation* OR termination* OR stopping OR ceas* OR stop OR discontinue* OR interrupt* OR holiday* OR withhold* OR dose-reduct* OR tapering*)) NOT (smok* OR tobacco*))

**Google Scholar**

anticonvulsant|antiepileptic|antidepressant|"anti convulsant|convulsive|epileptic|depressant" "stopping|interruption|cessation|holiday|termination|discontinue|withholding rules|guidelines|strategy" -smoking -tobacco

anticonvulsant|antiepileptic|antidepressant|'anti convulsant|convulsive|epileptic|depressant' 'stopping|interruption|cessation|holiday|termination|discontinue|withholding rules|guidelines|strategy' -smoking -tobacco
